# Editorial: Bridging the gap: physical manifestations and mental health in adolescents

**DOI:** 10.3389/fpsyg.2026.1838806

**Published:** 2026-04-17

**Authors:** Marika Orlandi, Martina Maria Mensi

**Affiliations:** 1 Department of Brain and Behavioral Sciences, Universita di Pavia, Pavia, Italy; 2 Child Neurology and Psychiatry Unit, IRCCS Mondino Foundation, Pavia, Italy

**Keywords:** adolescence, bullying, eating disorders, NSSI, prevention, psychopathology, suicidality, trauma

Adolescence is a critical developmental period marked by biological, psychological, and social changes. In this life span, mental health difficulties may emerge for the first time and be expressed through both emotional and bodily expressions of distress. Recently, concerns about youth mental health have grown and highlighted the need for a broader understanding of how suffering can be communicated through somatic complaints, behavioral dysregulations, and relational disruptions. The purpose of this Research Topic is to provide information on the different ways mental health can be expressed (as physical or behavioral problems) in the adolescent population and to provide insight into the complex relationship between mental health and physical/behavioral presentation in adolescents across multiple contexts. It will emphasize the importance of considering the relationship between subtle indicators of psychological distress (within young people) through their physical symptoms, relational difficulties, and contextual stress, by encouraging an integrated approach to early identification, prevention, and timely intervention.

This collection of articles focuses on how a person's body can serve as an expression of psychiatric distress, with several of the articles examining how adolescents express internal distress caused by mental health issues via physical or embodied ways. One article specifically looks at how eating disorders, Non-Suicidal Self-Injury (NSSI), and suicide attempts share a somatic as well as a behavioral component/pathway through which distress is expressed. In these cases, the body may be viewed as both an expression of distress and as a cry for help (Pratile et al.). Other research highlights the mediating role of cognitive flexibility in the relationship between social support and NSSI. Mental agility may turn protective relationships into concrete strengths against NSSI (Wang et al.). In these works, somatic and behavioral manifestations are important assessment points.

Relationship issues and the environment also help to connect the physical and mental dimensions. Early trauma and bullying are powerful triggers that connect social wounds to emotional and physical scars. Research shows that bullying has a negative relationship with self-esteem and multi-dimensional wellbeing (Song et al.), and serial mediation analyses show that bullying victimization exacerbates moral disengagement and relational harm (Bai et al.). The overall result of this research illustrates the circular pattern where peer

violence continues to affect the way individuals feel psychologically, but it also impacts them physically through symptoms of somatic tension, withdrawal, and decreased overall health in all areas of their lives.

In addition, this Research Topic explored the pathways of OCD symptom development amongst youth exposed to trauma (Demaria et al.), showing how adverse events create enduring intrusive patterns that spread to broader internalizing symptomatology. The co-occurrence of depression and anxiety among vocational high school students highlighted symptom bridges that may increase vulnerability to stress in highly demanding academic settings (He et al.). In parallel, internet addiction was associated with isolation, chronic constipation, smoking, and other physical complaints, creating clusters of vulnerability that are particularly relevant in digital-native generations (Fan et al.).

This Topic also stressed the importance of context in assessing physical and psychological distress. School and digital contexts, in fact, emerge as key amplifiers of distress. School climate emerges as a critical moderator, where supportive environments buffer BMI-depression associations that might otherwise increase (Jiang et al.). Sensory deprivation adds another layer, with touch deprivation in female adolescents disrupting semantic processing, cognition, and mental wellbeing, highlighting culture's role in shaping embodied experience (Sohail and Khattak). Finally, healthy personality traits and psychological flexibility protect youth facing transition stressors, positioning adaptive coping as a bridge from physical complaints to psychological resilience (Yang et al.).

All together, these 11 articles reframe physical manifestations not as comorbidities or artifacts, but as diagnostic portals revealing adolescent mental health landscapes. Those studies stressed the fact that the adolescent body expresses distress that demands interpretation across disciplines, and, crucially, physical manifestations are not mere comorbidities but signals of adolescent mental health needs.

This integrated perspective also carries practice implications. Primary care physicians who encounter recurrent somatic complaints should enhance their psychiatric knowledge and assess underlying internalizing patterns (Berbenyuk et al.), while teachers must address bullying not only as social aggression but also as a trigger for somatic distress. Mental health screening tools must go beyond verbal self-report to capture features that are not always mentioned in traditional questionnaires. Non-specialists such as coaches and teachers emerge as first-line detectors, trained to recognize embodied cries masked as truancy, academic decline, or physical inexplicabilities.

In conclusion, this Topic emphasizes the importance of inquiring into all somatic manifestations. Each of these articles bridges these different avenues of exploration, and clinicians, educators/teachers, and policymakers can use this information to develop interventions that best support adolescents through their complex developmental period. We invite readers to consider these articles together as a unified plea for integrated care that will ultimately change how we, as a society, respond to youth experiencing distress.

